# Comparison of Gunshot Entrance Morphologies Caused by .40-Caliber Smith & Wesson, .380-Caliber, and 9-mm Luger Bullets: A Finite Element Analysis Study

**DOI:** 10.1371/journal.pone.0111192

**Published:** 2014-10-24

**Authors:** Rodrigo Ivo Matoso, Alexandre Rodrigues Freire, Leonardo Soriano de Mello Santos, Eduardo Daruge Junior, Ana Claudia Rossi, Felippe Bevilacqua Prado

**Affiliations:** 1 Department of Forensic Dentistry, Piracicaba Dental School, State University of Campinas, FOP-UNICAMP, Piracicaba, São Paulo, Brazil; 2 Institute of Legal Medicine – IML-RR, Civil Police of Roraima, Boa Vista, Roraima, Brazil; 3 Department of Morphology, Piracicaba Dental School, State University of Campinas, FOP-UNICAMP, Piracicaba, São Paulo, Brazil; University of Illinois at Champaign-Urbana, United States of America

## Abstract

Firearms can cause fatal wounds, which can be identified by traces on or around the body. However, there are cases where neither the bullet nor gun is found at the crime scene. Ballistic research involving finite element models can reproduce computational biomechanical conditions, without compromising bioethics, as they involve no direct tests on animals or humans. This study aims to compare the morphologies of gunshot entrance holes caused by.40-caliber Smith & Wesson (S&W), .380-caliber, and 9×19-mm Luger bullets. A fully metal-jacketed.40 S&W projectile, a fully metal-jacketed.380 projectile, and a fully metal-jacketed 9×19-mm Luger projectile were computationally fired at the glabellar region of the finite element model from a distance of 10 cm, at perpendicular incidence. The results show different morphologies in the entrance holes produced by the three bullets, using the same skull at the same shot distance. The results and traits of the entrance holes are discussed. Finite element models allow feasible computational ballistic research, which may be useful to forensic experts when comparing and analyzing data related to gunshot wounds in the forehead.

## Introduction

Cadaver examination is performed to uncover the cause of death of an individual who has suffered accidental, suspicious, or violent death [1]–[3]. In this context, there are a variety of damaging agents that can cause organic changes that culminate in the cessation of human life, including mechanical, physical, chemical, and biological agents, and even those in mixed form. Among mechanical agents, firearm bullets are highlighted, as they are able to produce very harmful and lethal injuries [3], [4].

Portable firearms can be classified as short and long, and the importance of the study of short firearms is directly related to the fact that these are the predominant type used both for self-defense and to commit crimes [5].

Data concerning the region of injury in victims of violence examined at the Institute Oscar Freire in São Paulo (São Paulo, Brazil), in quantitative terms, point to the following percentages: head (40.7%), neck (7.0%), chest (12.6%); abdomen (9.6%), genital region (0.2%), upper limb (hand and upper segments respectively 12.82 and 10.48%), lower limbs (6.6% ) [6]. In the 1990 s, in Brazil, a total of 1,108,422 deaths from external causes occurred, and homicide ranked first, accounting for 33.3% (n = 369,068) of these deaths. Homicides involving firearms in 1991 exceeded 50% of all homicides in Brazil and at the end of that decade, in the year 2000, there was an increase in the contribution of firearms to homicide deaths [7]. Another study, also in Brazil, pointed out that in cases of homicide, the proportion of male mortality was greater than that found among other types of external causes, with a male/female ratio of 8.2. Among these deaths, 63.5% were committed using firearms [8]. Gunshot wounds were the most common causes of homicides and suicides, with no significant differences between 1993 and 1997 in the United States [9].

The study of wounds caused by firearm bullets is at the forefront of scientific research, in order to support the improvement of surgical techniques for the treatment or repair of ballistic trauma [10]–[12] and for the preparation and evaluation of personal safety equipment such as helmets and ballistic vests [13], [14], in addition to improving the forensic investigation of cases involving firearms, with or without fatalities [4], [15]–[17]. The appearance of a gunshot wound may not only indicate the bullet’s direction and trajectory, but also the type of ammunition and weapon used and the range of gunfire. Additionally, it may assist with identifying the manner of gunshot injury or death, with respect to it being accidental, homicidal, or suicidal in nature [18].

Some authors have tried to correlate, through quantitative and qualitative studies [17], [19]–[21] and through computational analysis [22]–[25], different aspects of gunshot wounds in the head inflicted by the most common types of handgun bullets. Wounds caused by firearm bullets can take varying shapes, due to the diversity of ammunition with respect to form and mass, speed, shooting distance, and angle of entry of the bullets, but few studies have established models for the ballistic analysis of gunshot wounds and biomechanical performance [25].

The scientific advent of finite element analysis has enabled the performance of dynamic studies and their applications in the automotive [26] and in the aircraft industries [27], for military [28] and medical purposes [29], for the investigation of the biomechanism in areas such as orthodontics [30], [31], implantology [32], [33], and blunt injuries [34], and as a new tool to aid in the forensic sciences [35]. Research studies that utilize finite element analysis on ballistics issues to reproduce gunshot effects in the human skull remain sparse. Scientific studies are needed to address the validation of this computational tool in correlating the morphology of bone injuries produced by the dynamic action of firearm bullets [24], [25].

The present study aims to compare the morphologies of gunshot entrance wounds in human frontal bone obtained in three dynamic shooting simulations (.40-caliber Smith & Wesson-S&W, .380-caliber, and 9×19-mm Luger bullets), using finite element analysis. The glabellar region was chosen to be the area of ballistic impact, because of the authors' experience in examining cases where assassins murder their victims with gunshots in the head (especially in the forehead or occipital region).

## Materials and Methods

This study was approved by the Committee for Ethics of Research of the State University of Campinas (Protocol number CEP-FOP-UNICAMP-066/2012).

The present research set the following shooting conditions (firing distance, firearm bullets and angle of impact) in order to simulate a hypothetical murder. It was considered a firearm (pistol) pointing perpendicularly to the glabella of the skull – which is a well-known anatomical landmark. In this case, both hypothetical “murderer” (represented by the bullet) and “victim” (skull) were considered as facing each other.

### 1. Bone surface acquisition

The authors used CT scan data from a human skull (GE HiSpeed NX/i CT scanner – General Electric, Denver, CO, USA), with a thickness of 0.25 mm, to obtain a three-dimensional (3D) surface of bone structures. The 3D skull surface was exported in stereolithographic (STL) format using the InVesalius 3.0b program (Center for Information Technology, CTI, Campinas, Brazil).

The skull was randomly chosen according to the following criteria: a) dry skull of unidentified individual (one sample of the laboratory of Anatomy, Piracicaba Dental School – State University of Campinas, Brazil); b) intact; no macroscopically visible bone pathology.

### 2. Construction of CAD geometry

The 3D CAD model of the human skull was built from the STL surface involving the upper third of the head and part of the middle third, in order to reproduce the frontal bone and its adjacent bones. This skull model, as well as the models of the three different types of ammunition (.40-caliber S&W, .380-caliber, and 9×19-mm Luger bullets), was constructed with freeform Non-Uniform Rational B-Splines (NURBS) surfaces by a reverse engineering method [35], using the 3D software Rhinoceros 5.0 (McNeel & Associates, USA).

These three different types of bullets were chosen based on their availability in Brazil: the 9×19-mm Luger is used by Brazilian military forces (Army, Navy, and Air Force) and federal police officers, the.40-caliber S&W is used by state military police officers and civil police officers, and the.380-caliber is permitted for use by the general public.

### 3. Finite element models

**Figure 1:**
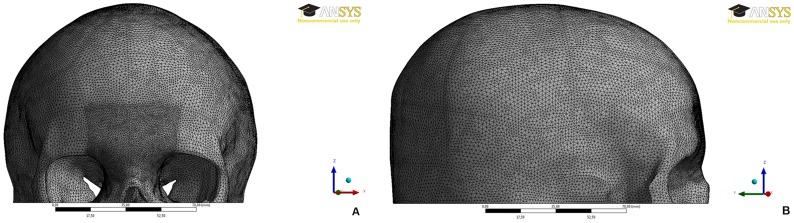
The finite element skull model: (A) FE model meshed with small tetrahedral elements particularly in the glabellar region. (B) Right view of the 3D-FE model.

**Figure 2:**
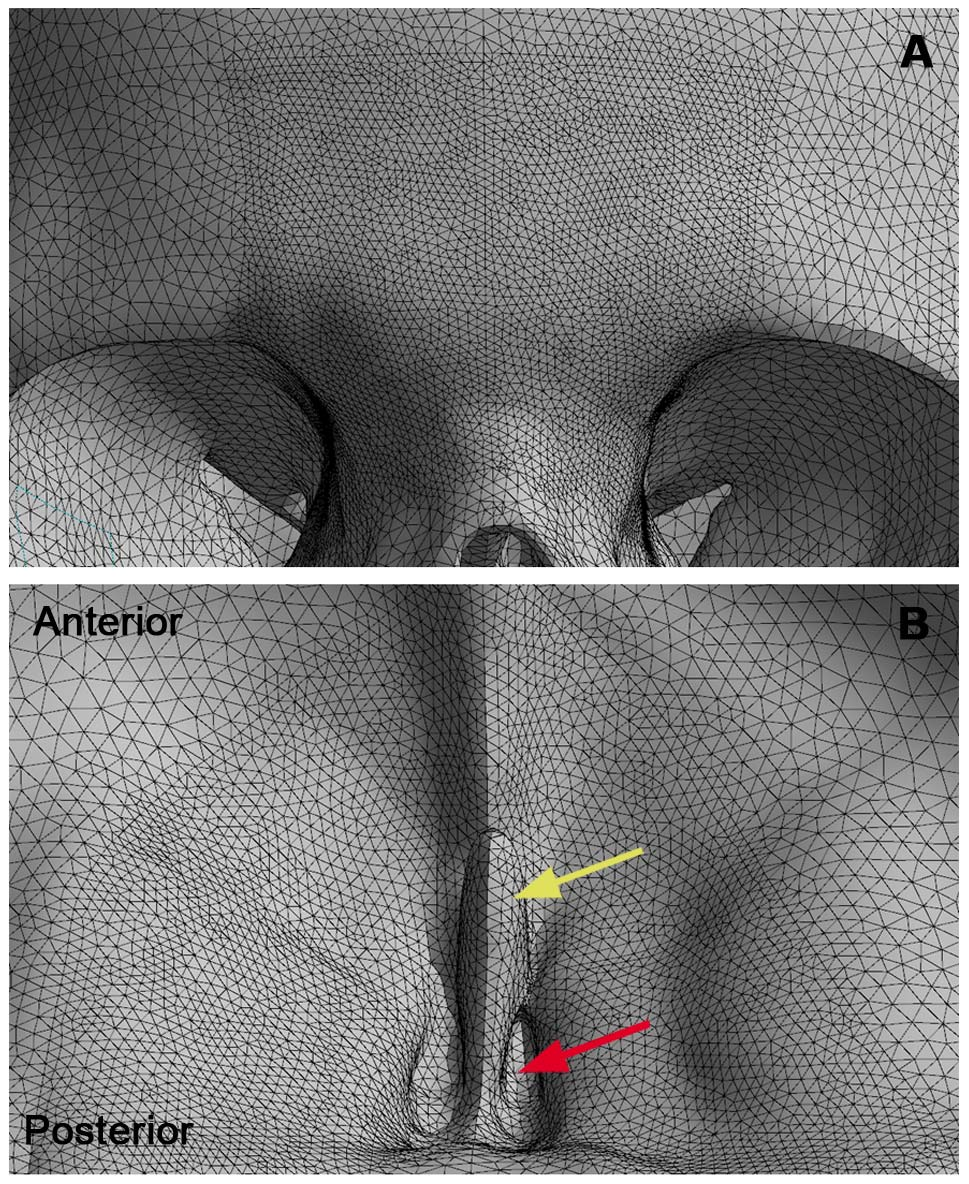
Refined mesh of the skull model. (A) Frontal view of the refined mesh in the ballistic impact area (glabellar region). (B) Internal view of the anterior cranial fossa with refined mesh in frontal bone and ethmoid bone (yellow arrow points to ethmoidal crest and red arrow points to cribiform plate).

The models (skull and bullets) were imported into ANSYS v.14 software (ANSYS, Inc., USA) for mesh generation with tetrahedral elements, which was refined in the glabellar region (Figure 1– A and B). The refinement was applied based on the element size reduction (see Table 1), only in the ballistic impact area (Figure 2– A and B), whose method was defined after element quality (q) evaluation by mesh metrics in ANSYS software. The mesh metrics showed an average q = 0.88 in whole skull model, where the best q = 1 and the worst q = 0. Thus, due to the high element quality in the skull, this quality was maintained at the glabellar region, changing only the element size.

**Table 1:** Number and average size of elements used in the finite element models (skull, ballistic impact area and bullets).

Finite elements	Skull	Ballistic impact area (refined area only)	FMJ RN.380 bullet		FMJ FP.40 S&W bullet		FMJ RN 9 mm Luger bullet	
			Jacket	Core	Jacket	Core	Jacket	Core
Number of elements	518812	306099	2635	4403	7183	14060	1958	4902
Average size of elements	1.5	0.8	1.2	1.2	1.2	1.2	1.2	1.2

The final mesh consisting of the system skull/bullet for each analysis was composed of 540,055 elements and 116,052 nodes in the shooting simulation with the fully metal-jacketed flat-point (FMJ FP).40-caliber S&W bullet; 525,850 elements and 113,088 nodes in the shooting simulation with the FMJ round-nosed (RN).380-caliber bullet; and 525,672 elements and 112,961 nodes in the shooting simulation with the FMJ RN 9×19-mm Luger bullet. The detailed mesh count of each structure is shown in Table 1.

### 4. Analysis configuration

Three computationally simulated gunshots were performed (one shot for each bullet) against the same skull model.

An explicit dynamics analysis was performed using Ansys v14 AUTODYN solver (Ansys, Inc.) for each shooting simulation with bullets of different calibers. The mechanical properties of bone structures were simplified based on a previous study involving craniomandibular mechanics in humans, in which the authors focused on the importance of the morphological performance of the human skull in response to mechanical stimuli [36]. Furthermore, the stress values in this shooting simulation were considered for comparison, due to the importance of the difference in caliber and the morphological characteristics of the aperture caused by bullet penetration. The mechanical properties of each material present in the bullets were selected according to the ANSYS database. The material failure properties of the bone structure were obtained from the database of MatWeb, LLC [37]. The mechanical properties of the finite element models (skull, .380-caliber bullet, 9×19-mm Luger bullet, and.40-caliber S&W bullet) are shown in Table 2. Thus, the erosion control, which defines the material failure criteria during the impact and bullet penetration in the skull, was based on the material failure properties according to the software configuration. This process is calculated by the program by combining the failure properties, the stress limits on geometry and the interaction between the models.

**Table 2:** Properties of the materials used in the finite element models.

Properties	Human bone^a^	FMJ RN.380 bullet		FMJ FP.40 S&W bullet		FMJ RN 9 mm Luger bullet	
		Jacket Cu^b^	Core Pb (99%)/Sb (1%)^c^	Jacket Cu^b^	Core Pb (99%)/Sb (1%)^c^	Jacket Cu^b^	Core Pb (99%)/Sb (1%)^c^
Young’s modulus (GPa)	14	115	14	115	14	115	14
Poisson’s ratio	0.3	0.3	0.38	0.3	0.38	0.3	0.38
Shear modulus (GPa)	5.3846	46	8.6	46	8.6	46	8.6
Bulk modulus (GPa)	11.667	129	–	129	–	129	–
Density (Kg/m3)	1850	8960	11340	8960	11340	8960	11340
Specific heat (J/Kg.°C)	440	383	124	383	124	383	124
Tensile Stress failure (GPa)**	0.133	–	–	–	–	–	–
Shear Stress failure (GPa)**	0.067	–	–	–	–	–	–

a Wroe et al [36].

b Copper (Cu) alloy UNS C23000 [37].

c 99% Lead (Pb)/1% Antimony (Sb) alloy UNS L52605 [37].

d Copper (Cu) alloy UNS C22000 [37].

**MatWeb Database.

For boundary conditions, displacement restrictions were applied along the three axes (x, y, z) in the lower plane section. The loading conditions were set in accordance with dynamic explicit analysis, involving the initial speed, the effect of gravity, and constraint conditions.

### 5. Conditions of velocity, shape, energy and shape of the bullets

The muzzle velocity conditions, mass, kinetic energy and the shape of the three bullets were selected according to data provided by a Brazilian manufacturer of weapons and ammunition (*Companhia Brasileira de Cartuchos*, *CBC*, Ribeirão Pires, Brazil), as seen in Table 3.

**Table 3:** Muzzle velocity, mass, energy and shape of each bullet in the computational simulation.

Bullets	Shape	Velocity (m/s)*	Mass (g)**	Energy (J)***
FMJ RN.380	Round nose	288	6.16	±256
FMJ FP.40 S&W	Flat point	300	11.66	±524
FMJ RN 9×19 mm Luger	Round nose	343	7.45	±440

*Meter/second; **gram; ***Joule.

### 6. Conditions of angles and distance

The angle of incidence set in the present study was a perpendicular angle formed between the long axis of the bullet and the glabella. The 3D-FE model of the skull was arranged considering the standard anatomical position set in the CT images (Frankfurt horizontal plane parallel to x-axis – anatomical position of the human skull). The shot trajectory was considered parallel to the ground for each dynamic simulation.

**Figure 3:**
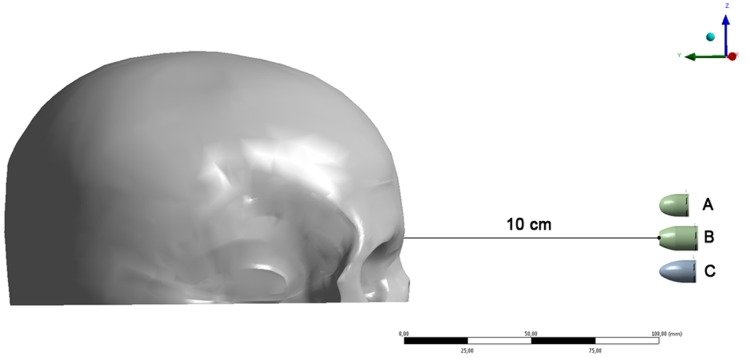
Right view of the firing distance at 10 cm: (A) .380-caliber bullet; (B) .40-caliber S&W bullet; (C) 9×19-mm Luger bullet. The dot indicates the starting point of each bullet, in each dynamic simulation.

The effects caused by air resistance were discarded due to the condition of very short-range shooting, which was set at 10 centimeters (cm) distant from the target (glabella). Theoretically, the distance of 10 cm corresponds to the virtual space between the handgun barrel of the three calibers (.380, .40 S&W, and 9×19-mm Luger) and the forehead of the 3D-FE model (Figure 3– A, B, and C).

### 7. Data analysis

Data were processed with the ANSYS AUTODYN solver, using a computer equipped with Intel Core i7-3770, 3.40 GHz, 32 GB RAM and NVIDIA Quadro 4000 video card, 1 GB.

Results were analyzed with respect to two aspects: the external morphology of the entrance holes in the frontal bone of the 3D-FE model and the equivalent von Mises stress distributed around each ballistic impact area.

## Results

In the present study, the skull model presented different wound geometry patterns.

### 1. Wound patterns

The data from the three computational gunshot wounds are listed in Table 4.

**Table 4:** External shape (morphology) seen after each shooting simulation.

Bullets	Wound type	Location	Shape (external)
FMJ RN.380	Entry	Glabella	Triangle, irregular
FMJ FP.40 S&W	Entry	Glabella	Round, irregular
FMJ RN 9×19 mm Luger	Entry	Glabella	Triangle, irregular

**Figure 4:**
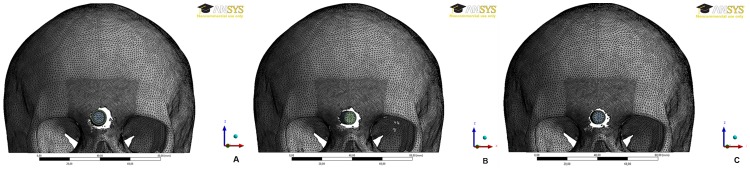
Simulated gunshot with: (A) .40-caliber S&W bullet; (B) 9×19-mm Luger bullet; (C) .380-caliber bullet.

Figure 4 (A, B and C) shows the moment of bullet penetration in the glabellar region.

**Figure 5:**
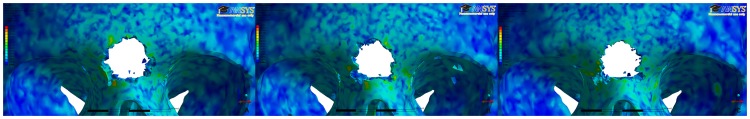
Morphologies of the entrance holes caused by the three computational gunshots. (A) Irregular round entrance hole (FMJ FP.40 S&W); (B) Irregular triangular entrance hole (FMJ RN 9×19-mm Luger); (C) Irregular triangular entrance hole (FMJ RN.380). Note that the wound seen in B exhibits an intermediate shape between those seen in A and C.

Figure 5 (A, B and C) shows the morphologies of the entrance holes caused by the three computational gunshots. The entrance hole morphology seen in Figure 5(A) resembles a round wound, while the one seen in Figure 5(C) resembles a triangular wound. Figure 5(B) shows an intermediate shape between a round (Fig. 5A) and triangular wound (Fig. 5C).

### 2. von Mises stress concerning each shot simulation

**Figure 6:**
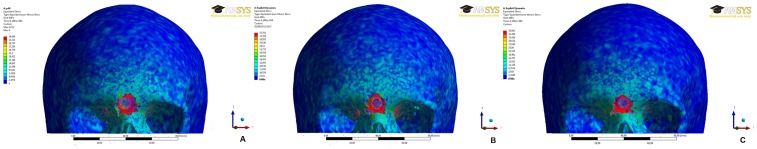
von Mises stress values in the glabellar region for each shot. (A) FMJ FP.40 S (B) FMJ RN 9×19-mm Luger; (C) FMJ RN.380.

The von Mises stress criterion is defined as stress caused by energy flow along a material that is receiving a load [38]. The stress distributes through the material, causing distortion until a critical resistance condition is reached, thereby causing failure. In this study, the critical stress resulted from energy flow caused by each bullet impact, and the consequent failure occurred in some regions of the skull model (e.g., glabellar region and orbital roofs).

von Mises stress is presented in Figure 6 (A, B and C), where the scale for the stress runs from the minimum stress value (blue) to the maximum stress value (red). The values of the maximum stresses were: 34,689 MPa for FMJ FP.40 S 33,781 MPa for FMJ RN 9×19 mm Luger and 38,983 MPa for FMJ RN.380. This information (see Table 5) is quite important in order to analyze and understand how the transferred energy of the bullet dynamically behaves within the gunshot injury. The floating red images correspond to those elements and nodes in which the effective stress was sufficient to cause complete failure in the connections of the mesh. The failure in the connections was seen where the bullet directly hit (glabellar region) and where the transferred energy was indirectly most affected (orbital roofs).

**Table 5:** Values of von Mises stress (MPa) for each shooting simulation.

Bullets	Dark blue (min)*	Green	Yellow	Red (max)**
FMJ FP.40 S&W	0–2,4778	14,867–17,345	24,778–27,256	32,211–34,689
FMJ RN 9×19 mm Luger	0–2,413	14,478–16,891	24,13–26,542	31,368–33,781
FMJ RN.380	0–2,7845	16,707–19,491	27,845–30,629	36,198–38,983

*Minimum; **Maximum.

### 3. Indirect gunshot wounds caused by stress distribution

**Figure 7:**
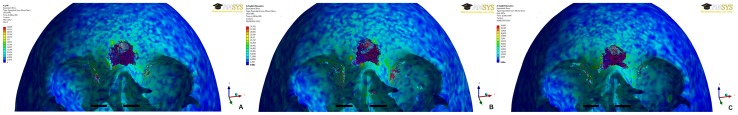
von Mises stress distribution and wounds in both orbital roofs of the skull model after each shooting simulation (A) FMJ FP.40 S (B) FMJ RN 9×19-mm Luger; (C) FMJ RN.380.

From the initial impact, additional wounds were observed. Figure 7 (A, B and C) shows show von Mises stress distribution and wounds in the orbital roof of both sides after each shooting simulation; these are considered indirect effects.

## Discussion

The authors are certain that several fatal or non-fatal gunshot injuries are daily examined by forensic experts around the world, but few computational studies have considered the characteristics of gunshot wounds in human head with regard to their medico-legal aspects [17], [22], [24], [39]–[43]. Human head models are mainly used for car crash evaluations and are not commonly used in forensic sciences [24].

The mechanism by which bullets injure living tissues has been extensively studied in animal models, human cadavers, and synthetic materials. However, these models are expensive and time-consuming, and whether these models represent situations within a living human body penetrated by bullets remains controversial [44], [45].

It is evident that the use of animals or human cadavers for experimental research has many limitations with regard to ethical and moral aspects [44], [46]. Moreover, once a ballistics experiment is carried out in animals or cadavers, it cannot, naturally, be reproduced in the same anatomical region in a subsequent trial to analyze other conditions.

Finite-element models of the skull are quite useful to reproduce and analyze different ways of injuring human bones, independent of the mechanical force applied. The interaction and behavior of damaging agents to the human head can be understood and the amount of energy or force measured using finite element analysis.

The results of dynamic simulations using finite element models may correlate with the degrees and patterns of biological tissue injury [44].

In the present study, the authors used the same finite element model of the human skull for computational simulations of gunshots with three different bullets. The glabellar region was the one chosen to be the area of ballistic impact, because of the authors' experience in examining cases where assassins murder their victims with gunshots in the head (especially in the forehead or occipital region). The tested skull model had frontal sinus, according to the CT-images, which was reproduced in the finite element mesh. Based on this information, the impacting zone had only the cortical bone (outer) and frontal sinus-cortical bone (inner). The orbital roofs had only a thin bone layer.

Different conditions (different skulls, longer or smaller firing distances, different designs and velocities of the bullets, angles etc) may exhibit different results from those presented in our study, or even have similar morphologies. This merits further studies about variations of the conditions stablished in this study.

The authors compared the external morphology of the entrance gunshot wounds caused by three bullets of different calibers, and the computational results showed different traits. The FMJ FP.40-caliber S&W bullet caused an irregular round wound, the FMJ RN 9×19-mm Luger bullet caused an irregular triangular wound, and the FMJ RN.380-caliber bullet caused an irregular triangular wound. As seen in Figure 5(B), the entrance hole shows an intermediate shape between 5(A) and 5(C). Considering that the simulations used the same FE skull model set at the same conditions (firing distance, axes, and directions), the different wound morphologies might be related to the kinetic energy of each bullet.

The kinetic energy information provided by the manufacturer (*CBC*, Ribeirão Pires, Brazil) (Table 3) indicated that the.40 caliber S&W bullet presented higher energy than the other two bullets (9×19-mm Luger and.380-caliber). This may explain why the rounded shape was more regular than the triangular shape caused by the 9-mm and.380-caliber shots (Figure 5– A, B and C). Additionally, the shapes of the bullets and the anatomy of the assessed frontal bone (flat and pneumatic bone) corresponded to the different gunshot entrance morphologies.

Table 5 shows the values of von Mises stress (MPa) of each simulated shot. According to these data, the bone tissue of the impacted area produced more resistance against the FMJ RN.380 bullet than against the FMJ FP.40 S&W and FMJ RN 9×19-mm Luger bullets. By comparing von Mises stress values between the two FMJ RN bullets (.380-caliber and 9×19-mm Luger), the 9×19-mm Luger bullet was able to penetrate the impacted area with lower resistance from the bone tissue.

In the comparison of the two bullets with the highest kinetic energies (.40-caliber S&W and 9×19-mm Luger), the von Mises stress was higher when the FMJ FP.40 S&W bullet was shot than in the FMJ RN 9×19 mm Luger bullet shooting simulation. This behavior may be associated with the difference in the shape of the nose of these two bullets: the.40-caliber S&W is a flat-point bullet and the 9×19-mm Luger is a round-nosed one. Because the flat-point surface of the FMJ FP.40 bullet is larger than the round-nosed surface of the FMJ RN 9×19-mm Luger bullet (see Figure 3– B and C), the von Mises stress was higher in the simulated shot with the flat-point bullet.

In the present study, the authors did not consider soft tissues (outer skin, meninges, brain) during skull modeling, neither did they reproduce the bullet’s gyroscopic properties (rotation, precession, and nutation), which may cause discrepancies between these results and real forensic data. Other finite element studies of gunshots did not consider those data either; however, they did provide useful information [22], [25], [44].

The gyroscopic properties of the simulated shots were not reproduced because the distance of 10 cm between each bullet and the target (glabella) was established as a short distance. However, future studies may reproduce these properties, especially with respect to longer firing distances.

Berryman *et al* [43] state that before any determination of bullet caliber from a gunshot defect to bone can be determined, a number of factors must be considered. These factors include the large variety of calibers available, some of which are very close or identical in diameter. Furthermore, bullets vary in shape and surface treatment, causing some to deform and produce a larger wound. The loss of gyroscopic stability may result in a more irregular or larger defect. Intermediate targets can result in a defect that is larger (from tumbling or deformation) or smaller (from fragmentation) than the bullet caliber. Another factor is a tangential shot that results in an irregularly shaped defect with portions that may be larger than the caliber. Finally, bullets that pass through an existing fracture may leave a defect that is smaller than the caliber. Our results showed the comparison of three morphologies of gunshot entrance wounds in a human skull model, in three dynamic simulations, using finite element analysis. The variables cited by Berryman *et al.* need further studies.

In conclusion, the present study proposed a finite element method to compare the results of three different gunshots using three different bullets at the same firing distance. The results showed different gunshot wound morphologies and their correlation to the amount of kinetic energy at the moment of impact, as expected in real shooting cases. The present study showed that the highest velocity bullet caused the rounded gunshot wound, while the lowest one caused an irregular triangular shaped wound. Finite element analysis is a practicable tool to be used in ballistics cases. However, further research is required to improve the methodology applied, in order to assist forensic experts in gunshot injury investigations.
